# Exploring Traditional and Cyberbullying Profiles in Omani Adolescents: Differences in Internalizing/Externalizing Symptoms, Prosocial Behaviors, and Academic Performance

**DOI:** 10.3390/ejihpe15060100

**Published:** 2025-06-04

**Authors:** Ahmed Al Saidi, Claudio Longobardi, Matteo Angelo Fabris, Sofia Mastrokoukou, Shanyan Lin

**Affiliations:** 1 Department of Behavioral Sciences Methodology, University of Valencia, 46010 Valencia, Spain; a.alsaidi@squ.edu.om; 2 Department of Child Health, Sultan Qaboos University, P.O. Box 50, Al-Khoudh P.C. 123, Oman; 3 Department of Psychology, University of Turin, 10123 Turin, Italy; matteoangelo.fabris@unito.it (M.A.F.); shanyan.lin@unito.it (S.L.); 4 Department of Political and Social Scineces, University of Salerno, 84084 Fisciano, Italy; smastrokoukou@unisa.it

**Keywords:** bullying victimization, cyberbullying, Omani adolescent, internalizing symptoms, externalizing symptoms, prosocial behaviors, academic performance

## Abstract

In the digital age, adolescents spend considerable time online, heightening their exposure to both cyberbullying and traditional bullying. However, few studies have investigated both forms of victimization simultaneously, particularly regarding their impact on adolescents in Middle Eastern countries such as Oman. This study used latent profile analysis to identify victimization profiles based on indicators of verbal, social, physical, and cyberbullying victimization. The participants included 1204 Omani students (604 girls, 50.2%; *M* = 14.04, *SD* = 0.20, aged 14–15). Three victimization profiles emerged: (1) non-victims (*n* = 989, 82.1%), (2) traditional victims (*n* = 156, 13.0%), and (3) dual victims (*n* = 59, 4.9%). The BCH approach revealed that adolescents in the non-victims profile, with the lowest levels of both traditional and cyberbullying victimization, reported the lowest levels of internalizing (*M* = 10.14, *SD* = 0.11) and externalizing symptoms (*M* = 10.36, *SD* = 0.10) and the highest academic performance (*M* = 4.59, *SD* = 0.02), whereas their prosocial behaviors were relatively low (*M* = 4.71, *SD* = 0.08). Adolescents in the traditional victims’ profile had moderate levels on nearly all outcomes. Adolescents in the dual victims’ profile, who experienced both traditional and cyberbullying, reported the highest levels of behavioral symptoms (*M_internalizing_* = 11.94, *SD_internalizing_* = 0.34; *M_externalizing_* = 12.81, *SD_externalizing_* = 0.38) and prosocial behaviors (*M* = 5.63, *SD* = 0.36), along with the lowest academic performance (*M* = 4.37, *SD* = 0.11). These findings underscore the need for culturally sensitive, multi-level interventions to protect Omani adolescents from both traditional and cyberbullying and to support their academic and psychosocial well-being.

## 1. Introduction

School is an important context for the cognitive, emotional, and relational development of adolescents (Fabris et al., 2023). However, different forms of violence can occur in this context, especially among peers (Longobardi et al., 2017, 2019a, 2021). Among the forms of peer victimization in the school context, one of the most widespread and that attracts particular attention in the educational and academic field is certainly bullying, which still represents a social emergency today (Fabris et al., 2021; Longobardi et al., 2019b; Thornton et al., 2024). We use the term bullying to refer to the intentional and repeated harming of a person where there is an actual or perceived power imbalance between the victim(s) and the perpetrator(s) (Hellström et al., 2021). By traditional bullying (TB), we mean a form of victimization that takes place in a real-life context, often face-to-face, and includes forms of physical victimization (hitting, kicking, pushing and shoving), verbal victimization (name-calling, teasing, and threats) or social victimization (spreading rumors and social isolation).

However, with the advent of new technologies, and, in particular, the proliferation of smartphones and easy access to the internet for adolescents, a new form of bullying has emerged in the online environment, known as cyberbullying (CB) (Fabris et al., 2022; Morese et al., 2024). Although TB and CB are conceptually similar and share different characteristics, the two forms of bullying also have distinctive features (H. J. Thomas et al., 2015). Traditional bullying typically occurs during school hours and in specific physical contexts. In contrast, cyberbullying transcends these boundaries, exposing victims to potential harm 24 h a day, 7 days a week. Furthermore, in traditional bullying, the victim knows the identity of their aggressors, whereas in cyberbullying, the identity of the attackers may be unknown to the victim, and the number of people witnessing the forms of victimization can be exponentially higher than in TB. Estimating the prevalence of (cyber)bullying victimization is not straightforward, as estimates can vary not only due to contextual and cultural differences, but especially due to different measurement strategies (Moore et al., 2017). Indeed, it is estimated that between 10 and 35 percent of adolescents have experienced traditional bullying (Li et al., 2024), while about 15 percent have experienced forms of victimization through cyberbullying (Jadambaa et al., 2019; Modecki et al., 2014). Furthermore, a recent meta-analysis (Li et al., 2024) estimated that one-third of adolescent victims of traditional bullying also tend to experience online victimization, while as many as two-thirds of CB victims also experience TB. This reflects the fact that TB is a strong predictor of CB and that a large percentage of cyber-victims are also victimized in an offline context. This is not surprising given that, at least in adolescence, the dynamics and quality of relationships experienced with peers in the offline environment appear to mirror and shape relationships in the online environment. Indeed, in the online world, adolescents might mainly keep in touch and interact with people from the real world, as in the case of class WhatsApp groups (Lin et al., 2025; Marengo et al., 2021). Therefore, it is possible that those who have poor relationships with peers in the physical/offline environment and are bullied by peers also do so in the online environment. However, data on the prevalence of bullying victimization and cyberbullying victimization are predominantly focused on Western cultures, especially Europe and the United States, while research has only recently focused on under-researched geographic areas, such as the Middle East.

In order to better understand how these forms of victimization are interrelated and influenced by broader factors, recent theoretical and cross-cultural studies offer valuable insights. Recent contributions to the literature have emphasized the importance of viewing traditional and cyberbullying as interconnected processes rather than as independent phenomena. Keith et al. (2025) proposed a socio-developmental trajectory in which adolescents involved in traditional bullying increasingly transfer aggressive behaviors to digital spaces, especially as peer interactions move online and group norms shift. This transition is facilitated by mechanisms such as digital disinhibition, anonymity, and the amplification of peer visibility through social media platforms. Similarly, C. Wang et al. (2024), through a longitudinal investigation, demonstrated that students who engage in offline aggression during middle school are more likely to become cyber aggressors or cyber victims by the end of high school, suggesting a fluid and reciprocal pathway between offline and online peer dynamics. These models point to dual victimization not as a simple accumulation of risks, but as a complex and evolving experience shaped by adolescents’ broader social ecology and digital literacy.

Moreover, cross-cultural studies have increasingly revealed that the prevalence, perception, and impact of bullying vary across regions because of differences in sociocultural norms, parental mediation, and digital access. Saleem et al. (2025), in a comparative study between Middle Eastern and South Asian adolescents, found that collectivist values, gender norms, and family involvement played a critical role in moderating the likelihood and psychological impact of cyberbullying victimization. Gallego-Jiménez and Rodríguez Otero (2025) further showed that school-based peer aggression in Southern European contexts differs in form and reporting patterns when compared with Northern European samples, particularly regarding cyberbullying. These findings highlight the need for localized research on dual victimization, particularly in under-studied areas like Oman, where cultural, religious, and technological frameworks may uniquely shape the experience of peer victimization. Incorporating such perspectives allows for a more ecologically valid and culturally sensitive interpretation of bullying dynamics and strengthens the theoretical contributions of this study within the global literature.

Beyond prevalence and definitions, an essential area of concern involves how victimization affects adolescents’ psychological and academic outcomes. There is a large body of research that shows how bullying and cyberbullying can affect students’ school adjustment and emotional well-being. By school adjustment, we mean a range of outcomes that affect not only academic performance (GPA), but also emotional–behavioral well-being at school (internalizing and externalizing symptoms) and better relationships with others (prosocial behavior). Several longitudinal and cross-sectional studies have documented the association between traditional bullying (Samara et al., 2021) and cyberbullying (Gardella et al., 2017; Wright, 2015) and poor academic performance in adolescence. It is possible that adolescents who are bullied tend to lose self-confidence and have low aspirations, and that depressive feelings can reduce concentration and energy to devote to learning. In addition, victimization can increase feelings of isolation and cause the young person to perceive the school environment as dangerous and frustrating, leading to high levels of absenteeism and truancy. It is clear that this situation is not optimal for the student’s academic journey and would significantly limit their learning opportunities. Although the literature suggests that both bullying and cyberbullying victimization are associated with poorer academic performance in adolescents, some data suggest that those who suffer from both forms of victimization perform worse than those who suffer from only one of the forms of victimization (Rostam-Abadi et al., 2024).

Another element that could characterize the school adjustment is prosocial behavior. By prosocial behavior, we mean social behavior that benefits other people or society as a whole and manifests itself in behaviors such as helping, sharing, donating, cooperating, and volunteering (Eisenberg et al., 2006). Prosocial behaviors are important in the school context because they allow children and adolescents to more easily build and establish positive relationships with peers, fostering greater integration into the class group (Longobardi et al., 2021), which, in turn, is a protective factor for victimization (Fu et al., 2023; Z. Wang et al., 2024). In this direction, Fu et al. (2023) found that low levels of prosocial behaviors in children and adolescents were directly and indirectly associated with the risk of TB, while no significant association was found with CB. However, other studies have indicated that adolescent victims of CB tend to act in a less prosocial way when they witness CB episodes as bystanders (Cao & Lin, 2015) and tend to become perpetrators themselves (Völlink et al., 2013). For example, it is possible that previous victimization experiences may lead to an increase in mistrust of others, as well as feelings of revenge and negative emotions among those affected, which could discourage prosocial behaviors in favor of more antisocial behaviors. Some data suggest that victims of CB are more likely to seek help from peers, parents, or teachers when they exhibit higher levels of prosocial behaviors (Holfeld & Mishna, 2019). Nevertheless, we must keep in mind that victims of CB tend to be isolated and have poor social skills (Marengo et al., 2024), which limits opportunities to develop higher levels of prosocial behaviors (Lin et al., 2024). However, not all studies agree. For example, Geng et al. (2022) found that adolescents who exhibit more altruistic behaviors online are also those who are more at risk of CB, whereas Kaiser et al. (2020) reported that although adolescents who were victims of cyberbullying exhibited higher prosocial behaviors than cyberbullies, they did so at a comparable level to their peers who were not involved in cyberbullying. In addition, several lines of evidence suggest that adolescents who are victims of bullying (Kokkinos & Kipritsi, 2012) or cyberbullying (Fabris et al., 2023) tend to show high levels of empathy compared to aggressors or uninvolved peers, which is an important precursor to prosocial behavior. These findings might appear counterintuitive, particularly given the elevated psychological distress observed in dual victims. One possible explanation is that, in collectivist contexts such as Oman, adolescents may adopt prosocial behaviors as a coping mechanism to restore social harmony or gain peer acceptance. This aligns with findings in other collectivist societies, such as Jordan (Shahrour et al., 2020), Saudi Arabia (AlBuhairan et al., 2017), and South Korea (Lee et al., 2022), where social cohesion and conformity are emphasized. Alternatively, the result could be influenced by social desirability biases typical in self-report measures, as suggested in studies from Iran and Pakistan (Saleem et al., 2025). These competing explanations call for further investigation using mixed-methods designs to clarify whether such prosocial tendencies reflect authentic behaviors, adaptive strategies, or reporting artifacts. These data seem to suggest that previous experiences of victimization may make adolescents more sensitive and attentive to the emotional needs and pleas for help of those in a situation of distress or danger. Thus, the link between prosocial behavior and victimization is important, but more research is needed to understand the nature and direction of this association.

Finally, bullying and cyberbullying are generally associated with an increase in psychological distress, which is associated with an increase in internalized symptoms (such as anxiety, depression, and suicidal ideation) and externalized symptoms (such as hyperactivity, conduct disorder, and substance use) (Prino et al., 2019). Research suggests that TB is associated with increased internalizing (Gini et al., 2018) or externalizing symptoms (Eastman et al., 2018; Marengo et al., 2018), while some evidence suggests that bullying victims report both symptoms (Eastman et al., 2018; Lin et al., 2024; Prino et al., 2019). However, a recent meta-analysis (Casper & Card, 2017) found that forms of peer victimization (both overt and relational) tended to be more strongly associated with internalizing symptoms than externalizing symptoms. This may be due to the fact that peer victimization during adolescence creates a greater sense of isolation from the group at a developmental stage when a sense of belonging to the group is crucial to adolescents’ emotional well-being, which also reduces internalizing symptoms, such as suicidal ideation (Longobardi et al., 2020). Peer victimization could thus lead the individual to reflect on their social failures, leading them to social withdrawal, a greater sense of loneliness, and fear of future victimization, thus undermining the possibility of building meaningful relationships with peers and developing appropriate social skills. Furthermore, a recent study found that internalizing symptoms are more associated with being a victim of bullying, whereas internalizing symptoms seem to better characterize the so-called bully-victims (Valente et al., 2024). Regarding victimization by CB, the literature shows similar results. Indeed, several studies show an association between CB victimization and an increase in internalizing symptoms (Elgar et al., 2014; Marengo et al., 2024) and externalizing symptoms (Fisher et al., 2016). In particular, a meta-analysis of longitudinal studies (Marciano et al., 2020) showed that not only does cyberbullying victimization predict both symptom areas, but that only internalizing symptoms predict the risk of becoming a victim of cyberbullying longitudinally. However, a more recent 3-year longitudinal study (Holfeld & Mishna, 2019) found, after controlling for TB victimization, that CB victimization was associated with both internalizing and externalizing symptoms and that both symptomatology (internalizing and externalizing) predicted CB victimization, not vice versa.

Although several studies have demonstrated an association between CB and TB victimization and measures of psychological well-being, few studies have focused on dual victimization, i.e., simultaneous victimization in offline and online contexts. These data seem to indicate that adolescents who are victimized in both contexts tend to have greater psychological distress than those who were only victimized in offline or only online (Ossa et al., 2023; Pengpid & Peltzer, 2023) and, in particular, report higher levels of internalizing symptoms (Chudal et al., 2021; Holfeld & Mishna, 2019; Pengpid & Peltzer, 2023; Schneider et al., 2012) and a greater tendency towards deviant behavior (K. J. Thomas & McCuddy, 2020) and risky behavior (Ossa et al., 2023; Pengpid & Peltzer, 2023; Rostam-Abadi et al., 2024).

### Aims of This Study

Although the Middle East region has been identified as a particularly vulnerable region for bullying in schools (Ran et al., 2023), much research is still needed to understand the prevalence of TB and CB in this geographical area and its potential impact on adolescents’ school performance and psychological adjustment. In particular, our study focuses on adolescent students in Oman, as this country is one of the Middle Eastern countries most affected by bullying in schools, according to the limited data available to us. In fact, according to the World Health Organization’s Global School-based Students Health Survey (GSHS) (see Abdalmaleki et al., 2022), bullying affects 47 percent of Omani students, while it affects 22.8 percent of students in the United Arab Emirates and 28 percent in Kuwait. Therefore, the aim of this cross-sectional study is to use latent profile analysis to identify victimization profiles based on indicators of verbal, social, physical, and cyberbullying victimization. In addition, we test whether there are differences in school adjustment (academic performance, prosocial behavior, and internalizing and externalizing symptoms) based on the identified clusters.

## 2. Materials and Methods

### 2.1. Participants and Procedure

The participants included 1204 students (grades 9 and 10) recruited from public schools in the capital of Oman (Muscat). There were 604 girls (50.2%) in this convenience sample. The average age of the sample was 14.04 (SD = 0.20), ranging from 14 to 15 years old. More detailed demographic information is provided in Table 1. There are no specific sample size guidelines for latent profile analysis (LPA), and a sample size between 300 and 1000 is generally considered sufficient for the commonly used fit-in indices to work adequately (Nylund-Gibson & Choi, 2018). The sample size in the current study slightly exceeded this range, which may have facilitated the identification of profiles with relatively low prevalence. The research plan was approved by the authors’ affiliated university (n. 0592463). This study strictly adhered to the ethical principles outlined in the Declaration of Helsinki, with all participants treated in accordance with ethical guidelines for research involving human subjects. Before data collection, the participants and their parents or legal guardians had to sign a consent form. The participants were assured of the confidentiality of the data and were informed that participation in this study was voluntary. They could refuse to participate in this study at any time or withdraw from this study if they felt uncomfortable answering any question.

### 2.2. Measures

#### 2.2.1. Adolescent Peer Relations Instrument (APRI; Parada, 2000)

Traditional bullying victimization was measured by the victimization part of the APRI (Parada, 2000). There are 18 items in this part that measure three dimensions of traditional bullying victimization, including verbal victimization (6 items, e.g., “I was teased by students saying things to me”), social victimization (6 items, e.g., “A student wouldn’t be friends with me because other people didn’t like me”), and physical victimization (6 items, e.g., “Students crashed into me on purpose as they walked by”). The students were required to rate all the items on a six-point Likert response scale (1 = Never, 6 = Every day). The sum of all the ratings was calculated and taken as the final score for traditional victimization, with a higher value indicating more victimization. The Cronbach’s alphas of the APRI were 0.80 (verbal victimization), 0.83(social victimization), and 0.76 (physical victimization).

#### 2.2.2. Revised Cyber Bullying Inventory-II (RCBI-II; Topcu & Erdur-Baker, 2018)

Cyberbullying victimization was measured by the RCBI-II (Topcu & Erdur-Baker, 2018). There are 10 items in the victimization section (beginning phrase “It happened to me” and “On the Internet”) of the RCBI-II (e.g., “sending embarrassing or hurtful messages”). The students were asked to rate these 10 items on a 4-point scale (1 = Never, 2 = Once, 3 = Twice or three times, 4 = More than three times). The final score for cyberbullying was calculated as the sum of all the ratings, which ranged from 10 to 40. The confirmatory factor analysis revealed an acceptable structural validity of the RCBI-II in this study: χ^2^/df = 4.81, RMSEA = 0.06, CFI = 0.99, TLI = 0.97, SRMR = 0.03. The Cronbach’s alpha of the RCBI-II victimization section was 0.84.

#### 2.2.3. The Strengths and Difficulties Questionnaire (SDQ; R. Goodman, 1997)

The self-reported version of the SDQ (R. Goodman, 1997) was used to measure the students’ internalizing symptoms, externalizing symptoms, and prosocial behaviors. In the SDQ, there are 25 items in 5 subscales, including emotional symptoms (5 items, e.g., “I am often unhappy, depressed or tearful”), peer relationship problems (5 items, e.g., “I am usually on my own”), behavioral problems (5 items, e.g., “I am often accused of lying or cheating”), hyperactivity (5 items, e.g., “I am easily distracted”), and prosocial behaviors (5 items, e.g., “I am helpful if someone is hurt, upset or feeling ill”). The students were asked to rate their behavioral symptoms and prosocial behaviors on a 3-point scale (0 = Not true, 1 = Somewhat true, and 2 = Certainly true). In line with the approach recommended by A. Goodman et al. (2010), the internalizing symptoms score (ranging from 0 to 20) was calculated by summing the peer relationship problems and emotional symptoms subscale scores. Similarly, the externalizing symptoms score (also ranging from 0 to 20) was calculated by combining the scores from the behavioral problems and hyperactivity subscales. The Cronbach’s alphas were 0.71 (internalizing symptoms), 0.70 (externalizing symptoms), and 0.76 (prosocial behaviors).

#### 2.2.4. Academic Performance

The teachers were asked to provide an assessment of each student’s overall academic performance by reporting their average grade across all school subjects. Each subject was evaluated using a standardized 5-point grading scale, where scores ranged from 1 to 5: 1 represented “Not Accepted”, 2 represented “Accepted”, 3 indicated “Good”, 4 represented “Very Good”, and 5 signified “Excellent”. Higher scores on this scale corresponded to higher levels of academic achievement, resulting in a cumulative average grade that reflected each student’s overall GPA.

### 2.3. Data Analysis

Data analysis was conducted using SPSS 29 and Mplus 8.3. To identify the profiles of traditional and cyberbullying victimization among Omani adolescents, we employed LPA with verbal victimization, social victimization, physical victimization, and cybervictimization as indicators. Models with varying profile numbers, from one to five, were analyzed to determine the optimal profile structure. Several criteria were used to evaluate model fit, including (1) Akaike information criterion (AIC), (2) Bayesian information criterion (BIC), (3) adjusted Bayesian information criterion (aBIC), (4) entropy (≥0.7, Reinecke, 2006), (5) the Lo–Mendell–Rubin adjusted likelihood ratio test, (6) the bootstrapped likelihood ratio test (BLRT), and (7) minimum profile size (≥5% of the total sample, Marsh et al., 2009). Substantive interpretability, utility, and classification diagnostics were also considered in selecting the final model (Nylund-Gibson & Choi, 2018). To compare behavioral symptoms and academic performance means across the latent profiles, we applied the BCH approach (Asparouhov & Muthén, 2014). This method was selected as optimal for evaluating mean differences between profiles, allowing for an overall chi-square test followed by multiple pairwise comparisons.

## 3. Results

### 3.1. The Identified Victimization Profiles 

To identify traditional and cyberbullying victimization profiles among Omani adolescents, latent profile analysis was conducted. Profile numbers ranging from 1 to 5 were tested, and the results are presented in Table 2. As shown in the table, the model with three profiles fits the data relatively well compared to the other models. Specifically, its LMR was significant, indicating that the three-profile model provided a better fit than the two-profile model. Its entropy was above 0.7, and the sample size in the smallest profile was above 50 and close to 5%. In addition, in the four-profile model, the LMR was nonsignificant, suggesting it did not improve upon the five-profile model. Therefore, the model with three profiles was selected as the final model.

**Figure 1:**
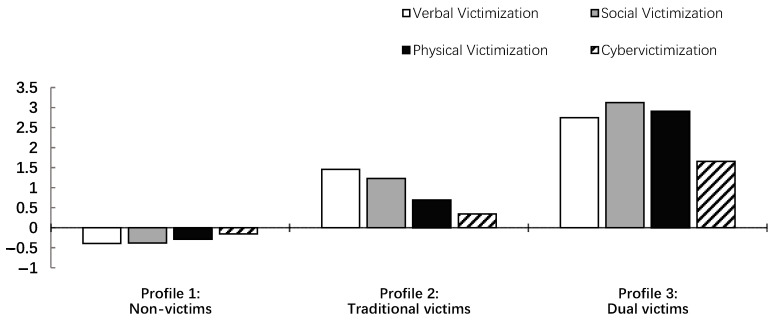
Z-scores of the indicators in each profile. Note, the longitudinal axis is the z-score of each indicator.

The z-scores of the indicators in each profile are illustrated in Figure 1 to better depict the characteristics of each group. As shown, Profile 1 (*n* = 989, 82.1%) represents the largest group, comprising students with all victimization z-scores below the average level. This profile was labeled as “non-victims”. Profile 2 (*n* = 156, 13.0%) was the second-largest group, where all indicators were positive, and the level of cybervictimization in this profile was relatively lower than the three forms of traditional victimization. This profile was labeled as “traditional victims”. Profile 3 (*n* = 59, 4.9%), as the smallest group, included students with the highest levels across all victimization types, including verbal, social, physical, and cybervictimization, relative to the other profiles, earning the label “dual victims”.

### 3.2. The Victimization Profiles and Profile Differences

To examine profile differences in internalizing and externalizing symptoms, prosocial behaviors, and academic performance, the BCH approach (Asparouhov & Muthén, 2014) was employed. The results are presented in Table 3. Significant differences were found between the profiles across all studied variables: internalizing symptoms (Wald’s χ^2^ = 25.32, df = 2, *p* < 0.001), externalizing symptoms (Wald’s χ^2^ = 44.16, df = 2, *p* < 0.001), prosocial behaviors (Wald’s χ^2^ = 7.68, df = 2, *p* < 0.05), and GPA (Wald’s χ^2^ = 14.19, df = 2, *p* < 0.01). Pairwise comparisons showed that the students in the non-victims profile (Profile 1) had the lowest levels of internalizing and externalizing symptoms, the lowest prosocial behaviors, and the highest GPA. The students in the traditional victims profile (Profile 2) exhibited moderate internalizing symptoms and had levels of externalizing symptoms and prosocial behaviors similar to Profile 1, with GPA similar to Profile 3 and relatively low. The dual victims profile (Profile 3) displayed the highest levels of internalizing and externalizing symptoms, the lowest academic performance, and the highest level of prosocial behaviors.

## 4. Discussion

Bullying and cyberbullying as forms of peer victimization are still a current social emergency. However, research on the prevalence and associated risk factors has mainly focused on Western countries, particularly Americans and Europeans, while only recently has interest in this topic begun to grow in previously under-researched geographical areas such as Middle Eastern countries, including Oman, in particular. Our study attempts to move in this direction and identify traditional and cyberbullying victimization profiles among Omani youth. We are not aware of any other similar studies in Oman.

From the analyses, three distinct profiles emerged that characterize the sample of adolescents examined, referred to as “non-victims” (profile 1), “traditional victims” (profile 2), and “dual victims” (profile 3). “Non-victims” represents the broadest profile and comprises around 82 percent of young people. This profile is characterized by adolescents who have experienced no or minimal victimization in any type of bullying or cyberbullying victimization. This finding seems to indicate that a large percentage of Omani adolescents are not exposed to any experience of bullying or cyberbullying, which is more positive than in other cultural contexts and seems to contradict the limited data available, according to which about half of Omani adolescents report at least one experience of peer victimization (Abdalmaleki et al., 2022). However, we must bear in mind that research on the prevalence of bullying in the cultural context of the Middle East is still underdeveloped, and cultural and methodological factors may play a role. For example, some scholars (Ginanjar et al., 2024) have pointed out that Arabic terms referring to bullying can have positive connotations and therefore do not always define bullying as a negative act. In Oman, the term bullying is known as “Suluk Audwani”, which describes a person who attacks another with their tongue or a weapon. Although this term evokes aggression towards another person, it seems to refer almost exclusively to a physical attack and underestimates the existence of forms of social and verbal bullying, which could have an impact on the prevalence of bullying reported by children and adolescents. In addition, Arab cultures tend to emphasize values such as courage, honor, and protecting one’s family and community, which may discourage adolescents from reporting bullying (Al Saadoon et al., 2024). The second largest group is the “traditional victims”, who represent 13 percent of the sample. This group is so named because the adolescents in this group have been exposed to peer victimization and, in particular, report high scores on all three forms of TB victimization (physical, verbal, and social) but low scores on CB victimization. Thus, the adolescent students in this group are characterized by having experienced traditional forms of bullying, but do not tend to report experiences of cyberbullying victimization. Finally, the third profile is labeled “dual victims” and represents approximately 5 percent of the sample involved. Adolescents in this sample have been exposed to all types of victimization and have high levels, including cyberbullying. Thus, these youth show high levels of TB victimization (physical, verbal, and social) and CB victimization. These data appear to be consistent with emerging trends in the literature, which suggest that traditional bullying is still more prevalent than cyberbullying (Jadambaa et al., 2019; Modecki et al., 2014). However, they also reflect the finding that the majority of adolescents who are victims of cyberbullying are simultaneously bullied in the offline world, while only a quarter of adolescents who experience TB are also victims of cyberbullying (Li et al., 2024).

In addition, our data attempted to understand the differences between the profiles in terms of school adjustment by measuring differences in the dimensions of academic performance, emotional and behavioral well-being (internalizing and externalizing symptoms), and prosocial behavior. Adolescents in Profile 2 (traditional victims) tended to have moderate levels of internalizing disorders and, similar to the dual victims, lower levels of academic achievement compared to the adolescents who were not exposed to bullying (Profile 1—non-victims). These data can be understood on the basis of previous literature. In particular, there is evidence that bullying victimization tends to be more strongly associated with internalizing symptoms than with externalizing symptoms (Casper & Card, 2017; Valente et al., 2024). It is possible that the experience of bullying increases a sense of low hope, isolation, and low self-esteem and fears of future victimization in victimized adolescents and thus promotes the occurrence of internalizing symptoms (Prino et al., 2019). In addition, the feeling of loneliness and reduced contact with peers could lead to unmet needs for belonging and closeness to the group, which increases the risk of internalizing symptoms (Casper & Card, 2017; Longobardi et al., 2020). The link between bullying victimization and internalizing symptoms may be particularly strong in Omani adolescents, considering that Oman is considered a collectivist culture. Therefore, harmony and group affiliation are highly valued in this cultural context, and experiences of victimization may reinforce perceptions of isolation and loneliness, making the link between victimization and internalizing symptoms particularly salient in this cultural context. However, there is evidence that adolescents with internalizing symptoms have characteristics that make them more likely to be targeted by aggressors, such as a greater tendency to isolate, greater introversion and poor social skills, which also make them less able to respond to bullying and defend themselves effectively (Valente et al., 2024). In addition, the Profile 2 (traditional victims) and Profile 3 (dual victims) subjects show low levels of academic performance compared to the Profile 1 subjects. These data are consistent with the literature, indicating that stress related to the experience of victimization can impair attention and motivation, thereby decreasing the likelihood of academic success (Gardella et al., 2017; Samara et al., 2021). In addition, it is possible that adolescents who are victimized by peers tend to avoid school in order to avoid interacting with the perpetrators of violence directed at them, thereby reducing not only the opportunities to build relationships with peers in the class group but also the opportunity for learning.

Finally, our study shows that the adolescents in Profile 3 (dual victims) are those who exhibit higher levels of psychological distress (they reported higher levels of internalizing and externalizing symptoms) and higher levels of prosocial behavior. The fact that the latter group reported higher levels of externalizing symptoms than Profile 2 may indicate that CB victimization tends to be equally associated with internalizing and externalizing symptoms (Fisher et al., 2016; Marciano et al., 2020). In general, the fact that the adolescents in Profile 3 reported higher levels of psychological symptoms is consistent with the literature indicating that adolescents exposed to both forms of victimization (CB and TB), rather than just one of the two, have poorer mental health outcomes (Pengpid & Peltzer, 2023). It is possible that adolescents exposed to both forms of victimization have difficulty finding a safe place as they are simultaneously victimized in offline and online contexts. In this way, it is possible that these adolescents may experience increased distress and feelings of loneliness and reduced opportunities to develop a sense of group belonging and more functional emotion regulation strategies, leading to an increase in psychological distress. In addition, the data show that adolescents who experience both forms of victimization exhibit higher levels of prosocial behavior. This finding warrants further examination, and we must keep in mind that the literature on the relationship between prosocial behavior and peer victimization is articulate, and the data are not always consistent. However, there is evidence that adolescents who report altruistic behavior online are at higher risk of being victims of cyberbullying (Geng et al., 2022). Other research instead suggests that adolescents who have experienced forms of peer victimization (both in online and offline contexts) tend to report more empathy (Fabris et al., 2023; Kokkinos & Kipritsi, 2012), which is a precursor to prosocial behavior.

In this regard, recent findings suggest that the elevated prosocial behaviors observed in dual victims may be interpreted through the lens of empathy-based responses. Sobol et al. (2025) highlight that some bullied adolescents, particularly those who are also bystanders or dual victims, may display heightened sensitivity to others’ suffering and adopt prosocial behaviors as a form of coping or self-protection. This behavioral pattern may serve to enhance social connectedness or reduce the risk of further victimization, especially in adolescents who feel vulnerable or isolated. Thus, the increased prosociality in dual victims may not simply reflect dispositional traits but rather a compensatory strategy shaped by their experiences of peer victimization. This behavior could also be encouraged by the particular cultural context in which belonging and group harmony, as well as prosocial behavior towards family and peers, are particularly valued. These authors hypothesize that previous victimization experiences have likely made victimized individuals more sensitive to the expression of others’ suffering, resulting in increased prosocial behaviors. However, further studies are needed to understand these differences and the mechanisms involved. Furthermore, in our study, higher levels of prosocial behavior were found only in the dual victims’ group, whereas the subjects in the traditional victims group showed similar levels to subjects in the non-victims group. Future studies should therefore investigate whether these differences are due to a more severe condition related to the presence of both forms of victimization (TB and CB) or whether the simultaneous presence of one form of victimization (especially online) determines these results.

## 5. Limitations of This Study and Future Research

Our study contributes to the understanding of the extent of the phenomenon of bullying/cyberbullying among adolescents in Oman, but our findings must be considered with the methodological limitations of this study in mind. As our study is cross-sectional, causal relationships between variables cannot be inferred from our analyses. For this reason, future replications in the context of longitudinal research projects are required. In addition, our research protocol included only quantitative self-report instruments. These instruments are valuable and widely used in this area of research, but they suffer from potential limitations in terms of social desirability, text comprehension, and the ability to correctly recall one’s memories. Future studies could include different measures and other observers. In addition, future studies could enrich the protocol with qualitative questions to elicit more subjective reflections from participants. Finally, although the sample is numerically sufficient for the analysis, it cannot be considered representative of the population of Omani adolescents. Future studies could therefore invest in recruiting representative samples and include both early and late adolescents. Finally, we suggest comparing the data from this sample with subjects of other ages (e.g., young adults or primary school children) or making comparisons with adolescents from other cultural contexts or adolescents with special needs to understand the possible role of cultural and socio-demographic variables.

### Practical Implications

Our study points to some possible practical implications, especially for teachers, educators, and psychologists dealing with adolescents. First, the fact that a significant percentage of adolescents have experienced forms of bullying and/or cyberbullying suggests the need to continue working in educational and school contexts, both at the level of prevention and intervention, in order to create more respectful and inclusive contexts and protect young victims. In addition, for young people with poor school adjustment, it is important to consider the possibility that forms of peer victimization are associated with it. On the other hand, for those who have been exposed to bullying and cyberbullying, it is important to consider how such experiences might be related to poor school adjustment and to intervene in the most vulnerable areas, such as psychological well-being, school performance, or interpersonal relationships. These findings suggest the importance of implementing differentiated intervention strategies tailored to the specific needs of the identified victim profiles. In particular, adolescents classified as dual victims—who report high levels of both traditional and cyber victimization along with increased psychological distress and elevated prosocial behavior—represent a high-risk group requiring targeted and multi-level interventions. For this subgroup, it is essential to design integrated programs that address emotional regulation, social skills training, and cognitive–behavioral support, with the goal of reducing internalizing/externalizing symptoms while promoting adaptive coping strategies. Furthermore, this study’s results highlight the need to extend intervention frameworks beyond the school setting to include the family context. Recent research (Amin et al., 2024; Chu & Chen, 2024) has demonstrated the protective role of family-based factors—such as parental monitoring of online activity, open communication about peer relationships, and parental modeling of emotional competence—in reducing the risk of both traditional and cyber victimization. Embedding parent-focused components in school-based programs could enhance the efficacy of interventions by fostering consistent messages and support structures across home and school environments. For instance, structured parental workshops on digital literacy and emotion-focused parenting may serve as critical levers in mitigating adolescent vulnerability to peer victimization. Finally, our data could provide information for the training and awareness-raising of professionals working with young people in different capacities. This implies the necessity for intersectoral collaboration among schools, families, and, potentially, digital platform stakeholders to co-construct evidence-based, systemic prevention models. Such collaborative approaches are increasingly supported in the literature as effective means of addressing the multifactorial nature of bullying and cyberbullying phenomena, especially in socio-cultural contexts where digital behaviors and parenting practices may differ.

## 6. Conclusions

Despite the aforementioned limitations, our study contributes to the current literature by providing insight into the extent of bullying and cyberbullying victimization among adolescents in Oman. In particular, our study shows that a proportion of adolescents are exposed to both forms of traditional bullying and cyberbullying (dual victims) and that this group of adolescents has poorer school adjustment than those who are only exposed to traditional bullying.

## Figures and Tables

**Table 1:** Participants’ characteristics of demography.

		Frequency	Percentage
Father education	Cannot read and write	26	2.2%
	Primary school	138	11.5%
	Secondary school	916	76.1%
	College	78	6.5%
	Postgraduate	46	3.8%
Mother education	Cannot read and write	8	0.7%
	Primary school	40	3.3%
	Secondary school	1044	86.7%
	College	111	9.2%
	Postgraduate	1	0.1%
Monthly income ^A^	325–500	44	3.7%
	501–900	313	26.0%
	901–1200	606	50.3%
	1201 and above	241	20.0%
Parent status	Live together	1199	99.6%
	Divorced	5	0.4%

Note: ^A^ The currency unit here is Omani Rials.

**Table 2:** Fit indices for bullying victimization profiles.

Number of Profiles	AIC	BIC	aBIC	Entropy	LMR	BLRT	Smallest Profile
One Profile	28,873.906	28,914.653	28,889.242	–	–	–	100% (*n* = 1204)
Two Profiles	26,341.997	26,408.212	26,366.918	0.989	2472.204 ***	2541.909 ***	0.15532 (*n* = 187)
Three Profiles	25,401.787	25,493.468	25,436.293	0.979	924.154 **	950.210 ***	0.04900 (*n* = 59)
Four Profiles	25,041.278	25,158.427	25,085.370	0.983	950.210	370.509 ***	0.01246 (*n* = 15)
Five Profiles	24,692.513	24,835.128	24,746.189	0.982	348.928	358.766 ***	0.01163 (*n* = 14)

Note: AIC = Akaike information criterion, BIC = Bayesian information criterion, aBIC = adjusted BIC, LMR = Lo–Mendell–Rubin adjusted likelihood ratio test, BLRT = bootstrapped likelihood ratio test; ** *p* < 0.01, *** *p* < 0.001.

**Table 3:** Mean differences in the studied variables between bullying victimization profiles.

	*M* (*SD*)			Mean Difference	
Outcomes	Profile 1: Non-Victims (*n* = 989)	Profile 2: Traditional Victims (*n* = 156)	Profile 3: Dual Victims (*n* = 59)	Overall Test Wald’s χ^2^ (*df* = 2)	Pairwise Comparison
SDQ_Internalizing symptoms	10.14 (0.11)	10.76 (0.26)	11.94 (0.34)	25.32 ***	P3 > P2 > P1
SDQ_Externalizing symptoms	10.36 (0.10)	9.94 (0.20)	12.81 (0.38)	44.16 ***	P3 > P2, P3 > P1, P2 ≈ P1
SDQ_Prosocial behaviors	4.71 (0.08)	4.94 (0.17)	5.63 (0.36)	7.68 *	P3 > P1, P1 ≈ P2, P2 ≈ P3
GPA	4.59 (0.02)	4.31 (0.08)	4.37 (0.11)	14.19 **	P1 > P2, P1 > P3, P2 ≈ P3

Note. P1 = Profile 1, P2 = Profile 2, P3 = Profile 3; * *p* < 0.05, ** *p* < 0.01, *** *p* < 0.001.

## Data Availability

The data that support the findings of this study are available from the corresponding author upon reasonable request.

## Abbreviations

The following abbreviations are used in this manuscript:

TB	Traditional Bullying
CB	Cyberbullying
LPA	Latent Profile Analysis
GSHS	Global School-based Students Health Survey
APRI	Adolescent Peer Relations Instrument
RCBI-II	Revised Cyberbullying Inventory-II
SDQ	Strengths and Difficulties Questionnaire
GPA	Grade Point Average
SPSS	Statistical Package for the Social Sciences
AIC	Akaike Information Criterion
BIC	Bayesian Information Criterion
aBIC	Adjusted Bayesian Information Criterion
LMR	Lo–Mendell–Rubin Adjusted Likelihood Ratio Test
